# Glaucoma, “the silent thief of sight”: patients’ perspectives and health seeking behaviour in Bauchi, northern Nigeria

**DOI:** 10.1186/s12886-016-0220-6

**Published:** 2016-04-21

**Authors:** Mohammed Mahdi Abdull, Clare Chandler, Clare Gilbert

**Affiliations:** Ophthalmology Department, Abubakar Tafawa Balewa University Teaching Hospital, PMB 0117, Bauchi, Bauchi State Nigeria; Department of Global Health and Development London School of Hygiene & Tropical Medicine, London, UK; International Centre for Eye Health, Department of Clinical Research, London School of Hygiene & Tropical Medicine, London, UK

**Keywords:** Glaucoma, Behaviour, Adherence, Treatment, Africa, Awareness, Acceptance, Traditional medicine

## Abstract

**Background:**

In Nigeria, glaucoma has a high prevalence and is the second cause of blindness among adults after cataract. People with glaucoma frequently present very late with advanced disease, and acceptance of and adherence to treatment is low. The purpose of the study was to explore how patients’ understand and respond to glaucoma in order develop an intervention to improve adherence to treatment.

**Method:**

Hospital based qualitative study. Six focus group discussions were held with patients with advanced disease and who had either undergone glaucoma surgery, were receiving medical treatment, or had neither surgery nor medical treatment. Two traditional healers who treat eye conditions were interviewed. Audio files were transcribed, translated into English and recurring themes coded and categorized as the impact of vision loss, and understandings of the disease and its management.

**Results:**

Visual loss impacted significantly on the lives of people with glaucoma in many ways. Many heard the term “glaucoma” for the first time during the study. Local terms to describe the symptoms included *Hawan jinin ido* (“hypertension of the eye”). Patients sought treatment in pharmacies, or with traditional healers who had different interpretations of glaucoma and its treatment to biomedical understandings. Cost and forgetfulness were the main reasons for low adherence to treatment while fear was a reason for not accepting surgery. Lack of money and negative staff attitudes were reasons for low follow up.

**Conclusion:**

Halting the progression of glaucoma is possible with treatment but the condition will remain a “silent thief of sight” in West Africa unless awareness, uptake of services and adherence to treatment improve. Understanding how glaucoma is locally conceptualised, lived with and responded to by patients is essential to aid the design of interventions to prevent glaucoma blindness in Africa. Findings have been used to adapt a motivational interviewing intervention, which is being evaluated in a clinical trial.

## Background

Glaucoma is the second commonest cause of blindness worldwide after cataract [1] and is the leading cause of irreversible blindness. In Nigeria, over one million people are estimated to be blind (0.78 % of the population), with 16.3 % attributed to glaucoma [2]. Together with its high prevalence, glaucoma in Africa is characterised by an early age of onset and aggressive disease course [3], Early presentation and treatment are essential to reduce the incidence of blindness. However, studies in Africa show that a high proportion of affected individuals only present once they are already blind in one or both eyes [3–10]. Once diagnosed, adherence to medical treatment is reported to be low [11] with high dropout rates [12, 13]. Surgical treatment is often not accepted or fails to control progression [11, 14, 15].

Understanding the way the symptoms and diagnosis of glaucoma are conceptualised lived with and responded to is essential to guide the design of interventions to increase early attendance for diagnosis and acceptance of treatment. Studies from around the world have identified the problem of low adherence or drop out from glaucoma treatment [16–19], being associated with poor knowledge of glaucoma [20–24], low levels of education [25, 26], black ethnicity [12, 27–31], worsening visual field loss [32], increasing age [28, 33, 34], lack of access to medication, inability to instil eye drops and forgetting to use the treatment [35–38]. Few studies have investigated this in African populations, although low awareness of the disease [39, 40], and fear and cost of treatment have been reported [41]. A diagnosis of glaucoma is often the first time a layperson encounters the disease [42–46].

The purpose of this study was to explore how patients understand and respond to glaucoma in Bauchi, northeast Nigeria: what they know about the disease, how they conceptualise its treatment and how they manage it, the family support and alternative sources of treatment. Traditional healers were interviewed to triangulate patients’ responses.

The study was undertaken in the eye clinic of Abubakar Tafawa Balewa University Teaching Hospital (ATBUTH) in Bauchi. The population are mainly subsistence farmers and levels of education are low. 70 % of the population live below the poverty line [47]. The catchment area of the hospital is approximately 4 million. Eye care is also provided in the State by another government hospital and a private mission clinic. Most expenses are covered by out of pocket payments by patients. Some government and private company workers have access to health insurance. Many people, particularly in rural areas, rely on traditional healers, who are mostly herbalists.

## Methods

Focus group discussions (FGDs) were undertaken with glaucoma patients identified in the eye department at ATBUTH. Traditional healers who treat patients with eye problems were also interviewed to elicit additional insights into patient perceptions and management of glaucoma. Topic guides were developed and used with probe questions to aid discussion as necessary.

### Participants

Six FGDs were undertaken. Three sub-groups of patients with late glaucoma in at least one eye were recruited: those who had undergone surgery, those on medication and those currently not on treatment, (Table 1). Late disease was defined as a visual acuity of less than counting fingers in at least one eye due to glaucoma. Eligible glaucoma patients were identified in the outpatient department using standard clinical methods. Further inclusion criteria included being resident in the state for at least 6 months, an ability to understand Hausa or English and willingness to participate. Separate FGDs were held with men and women to encourage open responses rather than to elicit gender differences. Escorts of the same gender were permitted to attend. Patients understood that their responses would be used for intervention design and completion of academic studies by the researcher. The FGDs took place in a quiet room in the hospital and each FGD comprised 7–11 patients and 1–3 escorts, and discussions typically lasted for 1–2 hours. Two traditional healers, who were identified by asking patients who they had visited, were interviewed in their homes, which also doubled as their places of work. Interviews and FGDs were conducted by a female social scientist experienced in counselling and interviews. Discussions were audio recorded with permission from the group. A note taker recorded additional observations. The lead investigator (MA, a male) was present only during interviews with the traditional healers who were both male.

**Table 1:** Glaucoma patient study sub-groups and topics

Sub-group	Topics of particular interest
Late glaucoma patients who had undergone surgery	To gain insights into what motivated them to accept surgery. What their fears were prior to their decision and how they overcame them.
Late glaucoma patients who were on medication	To explore the motivation behind their reported adherence to medication while facing a real threat to their vision. To explore coping techniques used to ensure availability of their drugs and adherence. To explore if they were ever offered surgery to find out why they did not accept it.
Late glaucoma patients who had not undergone surgery and were not on medication	To explore reasons for presenting very late to hospital, accepted surgery or medication. To try to understand the barriers they could not overcome.

### Data management

All records from discussions and interviews, including written notes, audio files and summaries of each event were linked by unique identifiers and stored securely. Audio files were transcribed verbatim into Hausa and proofread against the audio file by both the interviewer and the lead researcher for accuracy. Transcripts were then translated from Hausa into English and rechecked by the researcher (MA). A meaning-based translation approach was used: where there was no terminology for a word in English it remained in Hausa. All audio files, transcripts, translations and summary notes were uploaded into Nvivo for coding and analysis. The analysis was undertaken using the Hausa transcript by the researcher who is bilingual in Hausa and English. Relevant sections of transcripts and observers’ notes were used in the analysis to explore recurring themes, which were coded in NVivo software (QSR International). These were categorized by the researcher in consultation with the interviewer as an ongoing process through the fieldwork, according to the topics of interest i.e.understanding of glaucoma, adherence to medication, their views on traditional healers, acceptance of surgery and follow up, family support and problems faced because of glaucoma. The analysis followed a meaning-based approach, whereby themes were seen as emergent from the interaction between researchers, their questions and research subjects. Only themes considered strongly represented in the data are presented. Samples of the interviews were read by CC and CG in order to converge on the most salient themes to answer the research questions. Relevant quotes presented in this paper were extracted from the English translation.

## Results

Sixty-six patients participated, approximately half of whom were male. Approximately a fifth were escorts who were usually close relatives or spouses who live with and care for the patients. Many patients were happy to talk about their condition and it seemed important to them that someone cared enough to listen. None refused invitation to participate and participants were not invited back to provide feedback on the findings.

### Understanding of glaucoma

Despite having late stage disease, many said they heard the term “glaucoma” for the first time during this study. Those familiar with the term had heard it from hospital staff after diagnosis. However, people were familiar with the symptoms, but not universally.

The peripheral field loss characteristic of glaucoma was graphically described by some, as *Taka shanya* which describes stumbling when walking looking straight ahead and *Ciwon dundumi* which means “groping illness”, a common term in areas where onchocerciasis (“river blindness”) is endemic. Some used more general terms such as *Hazon ido*, which means cloudy vision, and *Yanar ido,* which is commonly used to describe cataract. Some attributed the symptoms to *Hawan jinin ido,* which literally means hypertension of the eye, or *Amosanin kai*, which means dandruff of the scalp.

Given the multiple local constructs relating to the single biomedical category of glaucoma, it is not surprising that multiple causes of these symptoms were reported.

“It is caused by high blood pressure”. FGD 01-M52

“Bleeding in the eye as a result of accident can bring about glaucoma”. FGD 02-F52

“Being possessed by evil spirits which attack the eye”. FGD 05-M33

“Apollo infection can turn into glaucoma”. FGD 03-M43

Apollo is a local name for a highly infectious form of viral keratoconjunctivitis, which occurs in epidemics in West Africa. Other causes included eating processed food, exposure to cold, prolonged crying/mourning, and frequent exposure to smoke from fires. However, many respondents admitted that they did not know what causes this eye condition.

### Perceived threats because of blindness

Visual loss from glaucoma leads to disruption in everyday life and can have a major impact on activities, social standing and aspirations, which were described vividly by some. Table 2 describes the impact of blindness from glaucoma according to Khadka, and adherence to treatment regrouped according to Newman-Casey [48, 49].

**Table 2:** Examples of participant responses on specific topics about glaucoma

Impact of glaucoma and blindness from glaucoma
Emotional well-being
“This sickness has affected our ambition and killed our dreams”. FGD 06-M47
“As human nature, relatives, friends and neighbours sometimes avoid us. In fact the relationship is not the same again”. FGD 03-M62
“Our relatives are trying their best but sometimes they get tired”. FGD 06-F69
“I wanted to study so much but I got this disease at secondary school. I am not happy about it since I was one of the best students in school. I still dream about school”. FGD 02-F40
Social issues
“This sickness has also affected our roles and the status we occupy at home and in society generally”. FGD 06-M47
“The sickness affects our social activities. For example, we cannot attend some social gatherings”. FGD 06-F50
Economic issues
“I cannot do the work I used to do before the onset of this sickness”. FGD 03-M30
“I am a professional and I cannot do my job now. I used to farm very well but now I cannot do it. Lack of sight has deprived me of many things” FGD 03-M51
Resilience
“When one is blind, he/she just has to tolerate and bear certain things”. FGD 01-F22
Impact on family and relationships
“Relations show concern and care to our sickness”. FGD 01-F40
“My sickness affects every activity my relatives are engaged in. For example they have left their farms, business, work and little ones alone just to be with us here in the hospital”. FGD 04-F50
“Family and relatives help us in most of our chores, but they may not do everything the way we want it done”. FGD 04-F52
Seeking treatment
“My neighbour prescribed my eye drop for me when I told him I have glaucoma because he also has the same illness. FGD 01-M57
“I don’t think glaucoma or any other eye problem has traditional cure. This is because traditional healers don’t have equipment to test any eye disease”. FGD 04-M26
“I once use traditional herb, but there was no improvement”
“Traditional healers use one herb to treat many different ailments, so they can be very dangerous”.FGD 01-F45
“I once met a traditional leader who told me my problem was glaucoma. He wanted to operate on my eye but my colleagues warned me and said if the surgery does not work, where will you see the man to complain or sue him in the court when my eyes go blind? So I opted out and refused the operation” FGD 05-M32.
Adherence to treatment
Forgetfulness
“We try our best to use the drugs as prescribed, but it is natural as human beings to forget once in a while”. FGD 03-F35
Beliefs about medication / side effects
“I don’t intend to use the drugs for too long because I believe that long term usage can cause complications for me” FGD 01-M54
Medication of no benefit
“As a blind person, I no longer use the drugs because I feel they are not useful to me since I cannot see”.
“There was a time I defaulted because the drugs were not working for me”. FGD 01-M54
Satisfaction with medication
“I am happy using eye drops but would prefer to be given spectacles” FGD 05-M49
“Drugs have helped to prevent the remaining part of my eye from complete blindness”. FGD 05-M49
Acceptance of surgery
“I know people that have undergone glaucoma surgery and they are seeing better now”.
“My sister was completely blind, but doctor X performed surgery and it was successful, but I don’t know if her case was glaucoma”. FGD 06-F69
“I have had surgery and can testify that I can see better after the surgery than I was seeing before the surgery too”. FGD 01-M43
“I will accept an offer of surgery because from the information we received about glaucoma, surgery is the best option to avoid blindness”.FGD 04 M38
“I have not rejected an offer of surgery from the hospital, but financial constraints made me delay the surgery”. FGD 04-F45
Follow up
“Keeping follow up appointments will help us especially when we have problems with the drug or in an instance of side effects”. FGD 04 M38
“You will come very early in the morning waiting to see the doctor, but you spend the whole day because they are seeing people who came after you, even if they are their staff or family. This is not fair and I don’t want to quarrel again with anyone”. FGD 06-F62
Communicating about glaucoma
“I can inform others about glaucoma based on the information I have received during this discussion”. FGD 06-M47

Coping with loss of vision was a major issue. Some were able to cope with family support while others harboured resentment. Loss of job, self esteem or profession were major issues. A young man had this to say.

“This sickness has affected our ambition and killed our dreams”. FGD 06-M47

Glaucoma patients described different forms of support from family and friends, and some spoke of the burden their condition placed upon these carers. Sometimes the responses of others were not supportive:

“My neighbour was happy I was on my way to blindness”. FGD 06-F56

### Seeking treatment

Patients sought treatment in a variety of places. Some went to pharmacies to purchase eye drops or seek advice about treatment while others consulted friends or neighbours. Some respondents described visiting traditional healers before coming to the hospital and there were a range of responses (Table 2).

“Traditional medicine is trial and error”. FGD 04-M31

“It is possible to get cure from traditional herbs because most of the modern medicine we use are processed from traditional herbs”. FGD 03-M52

### In-depth interviews with traditional healers

Traditional healers were often visited first by patients before attending hospital while others visited healers as a last resort.

“Most patients come to us after losing hope in the medications offered by the hospitals they visited” IDI 02–1

One traditional healer had strong views about the causes of glaucoma and how it should and should not be treated:

“Glaucoma and cataract are like younger and older brothers. They are caused by the bite of flies known as ‘fuleria’. Once bitten by the carrier fly, a person is afflicted by the ailment and can carry the germ for up to 3 years or 4 years or sometimes for up to 5 years before it affects the mucus of the eye….once it starts the poison now changes the mucus to something like groundnut oil and becomes a cataract. If it is becoming glaucoma it doesn’t separate - it mixes to look like engine oil that has lasted too long in a motorcycle then it liquefies.” IDI 01–1

“My medicine makes the mucus dissolve like ice in water. It burns out the oily layer formed in the eye and the patient is cured…glaucoma cannot be treated by surgical operation. There is no doctor on the surface of this world that can convince me of that.” ID I01-1

### Adherence to medication

Most patients administered the drops themselves while a few had help from carers. Many elderly patients said they rely on their children, and women often relied on their husbands to buy the drugs, having little or no control over when they would be available. Most respondents admitted to not being very adherent to their treatment regimes (Table 2), giving a range reasons including lack of information about how to take their treatment. For example,

“I was not told whether to continue or just administer the drug when in need so I use it as occasion demands.” FGD 06-F42.

Adherence to medication was also difficult due to costs and also availability, particularly for those living in rural areas who ended up purchasing drugs from other sources, such as local pharmacies, where the potency of drugs cannot be guaranteed.

The second commonest reason was forgetfulness or preferring other treatment.

“We sometimes run short of money to buy the drugs”. FGD 03-M61

“Sometimes the drugs are difficult to get from the pharmacy shops, especially when the hospital pharmacy runs short of the drugs. Sometimes the drugs we buy outside the hospital pharmacy are not as effective as the ones we buy at the hospital”. FGD 02-M45

“I try to take the drugs as prescribed by the doctors, but sometimes I do forget”. FGD 05-F59

Quite a few patients said that they only use their drugs if they develop symptoms. Even though glaucoma has few symptoms apart from loss of vision, some patients claimed to know when the pressure is high as they develop headaches. Some patients understood that treatment preserved sight whereas others no longer used medication, as they were already blind or had poor adherence as they thought treatment should improve their vision. Some participants did not adhere to medication because of side effects such as headaches, hiccoughs, red eyes, gritty eyes and poor sleep. Some feared complications later in life.

“I use the drugs but when I feel better I reduce the frequency of using the drugs”. FGD 02-M43

“I have used the drugs for some time but there is no change in my vision, so I don’t know whether the drug is working or not”. FGD 06-F41

### Acceptance of surgery

Most reasons for accepting or rejecting surgery were based on the experiences of others. Some patients reported that what they need is confidence in the doctor’s advice and abilities, and a reliable, efficient service.

Fear of blindness, cost of surgery and the fear of surgery itself were reasons for not accepting surgery in some.

“I will accept surgery but the doctor must be sure that surgery is the best treatment for me”. FGD 03-M35

“There are difficulties in going to the hospital. One could spend the whole day without seeing the doctor then will be told to come back in a week or a month after spending transportation money….” FGD 06-F55

“I am afraid of the surgery”. FGD 06-F65

### Follow up

There was general agreement that follow up is important because it will help the doctor address their problems and monitor progress of their disease. Many patients did not specify why they defaulted from follow up, explaining that the reasons were beyond their control, for those that did, lack of money, and negative attitudes of some staff which may reflect their views about the inefficiency of the service was a factor (Table 2).

“You will come very early in the morning waiting to see the doctor, but you spend the whole day because they are seeing people who came after you, even if they are their staff or family. This is not fair and I don’t want to quarrel again with anyone”. FGD 06-F62

### Communicating about glaucoma

Most participants said that trained staff were in the best position to educate people about glaucoma. More than half said that glaucoma patients were in the best position to enlighten others. Most respondents said that the best means of creating awareness about glaucoma in the general population is through the radio, television and health talks whilst others mentioned household heads, community leaders, health organisations and government agencies (Table 2).

## Discussion

This study explored patients’ understanding of glaucoma, their health seeking behaviour and how they cope with the disease and its treatment in everyday life in Bauchi, Nigeria. When illness strikes it is common in all societies to ask, ‘why me? why now?’ [50]. These questions are challenging in glaucoma even with biomedical knowledge, as the disease does not yet have a clear underlying cause. Lay explanations in this study included exposure to certain foods, wood smoke and crying, or supernatural or fatalistic causes, implying that this is what God had ordained for them. Eye care providers need to be aware of these views, and be respectful and considerate [50].

In this study some participants visited traditional healers either when they first noticed problems with their vision or once they had given up hope in allopathic services. From the author’s experience, traditional healers are respected and trusted in northern Nigeria, having considerable influence on patients’ and their carers’ decisions: indeed, the word of the traditional healer is often believed above that of medical doctors. In this study some participants believed that traditional healers could cure their glaucoma. However, other participants were of the view that traditional healers could not be relied upon as they lack the ability to make an accurate diagnosis. This is supported by the interview with one healer who appeared to confuse glaucoma with onchocerciasis, saying that it followed the bite of a fly. This healer had confidence in his own treatment and was emphatic that surgery was not appropriate for glaucoma. To our knowledge this is the first study to interview traditional healers about glaucoma.

Understandings about glaucoma varied, with some patients having very good knowledge of the condition and its treatment whilst others had no awareness, hearing details for the first time during the study. The lack of a universally understood name for glaucoma in the majority Hausa community reflects that it is not a well-known disease. It is important to have a common nomenclature that describes glaucoma so that it is not mistaken for other treatable conditions such as cataract, for example, which can have tragic consequences. In this study the most fitting descriptions that correlate with the biomedical definition of glaucoma were *hawan jinin ido* (hypertension of the eye), and *taka shanya* (stumbling on objects without having seen them). The first derives from patients’ understanding of hypertension which is quite common in Nigeria [51], while the second describes peripheral visual field loss typical of glaucoma and onchocerciasis. These terms could be used to create awareness in the community and in patient education, gradually introducing and popularizing the term glaucoma, as has been suggested in Ghana [52]. Lack of knowledge of glaucoma is commonly reported from developing countries such as Ethiopia [39] and India [44], but awareness can also be low in more developed societies such as Brazil [53] and in the USA [54]. Greater awareness has been reported amongst those with higher levels of education [29] and is associated with more appropriate eye care seeking behaviour [42, 43].

In the Netherlands improving knowledge was considered best delivered by qualified staff, or representatives of glaucoma patients’ societies, which requires trust in health professionals [55]. In our study some participants recommended peer-to-peer methods as a means of encouraging others with glaucoma to accept treatment, but there is the potential for misunderstandings to be perpetuated. Glaucoma support groups or clubs could also be formed, as have been established elsewhere, to aid interaction among patients with a view to improving awareness, knowledge and adherence to treatment [56]. Glaucoma support groups can also advocate for better availability of affordable medication, and encourage others to be assessed for glaucoma [56].

In our study some participants did not understand that the purpose of treatment is to preserve existing vision rather restore sight, which is one explanation for low acceptance and adherence as there was no perceived benefit. Other reasons included forgetfulness, side effects and fear that long-term use would accelerate blindness. Amongst those who did want to use medication cost, lack of medication in hospital pharmacies and difficulty in instilling eye drops were described. In this setting as elsewhere, these are legitimate challenges. Similar findings have been reported from Jamaica and the Netherlands [27, 57].

Fear of a poor outcome was an important reason why patients do not accept surgery. Combined cataract and glaucoma surgery is being advocated for Africa, even if there are only minimal lens opacities, so that patients experience some improvement in vision after glaucoma surgery [58]. Fear can be allayed in different ways: the HIV/AIDS program in Ethiopia used entertainment education to help patients cope with fear and to promote preventive actions [59].

Adherence to eye drops requires using the drug at the right time as well as delivering the drug into the eye, which can be challenging [49]. Several studies have shown deficiencies in self-administration of eye drops, including in Baltimore where 29 % of glaucoma patients were unable to instil their drops [60]. It is, therefore, essential that patients use reliable systems to remind them when to use their drops, and are trained to instil drops themselves, or others administer the drops. The extended family system in Nigeria would make the latter a feasible solution.

Adherence to medication in all chronic diseases is a challenge, and many interventions have been evaluated and could be adapted locally. Interventions include motivational strategies for hypertension [61], and education or counselling for latent tuberculosis [62]. Intensive reminders and ‘implementation intention’ interventions appear promising in antiepileptic mediations [63] and patient support and education for antiretroviral therapy [64]. Although complex interventions consisting of patient education combined with personalised behavioural change interventions, including tailoring daily routines to promote adherence to eye drops, may improve adherence in glaucoma there is insufficient evidence to recommend a particular intervention [65]. A major review of use of education to improve adherence demonstrated that educational interventions led to a significant improvement in medication adherence or a trend in improvement [66].

In our study several participants complained of inefficient services with long waiting times and poor attitudes of staff, which made them reluctant to attend for follow up. This needs to be addressed by reorienting and educating staff to be more patient friendly and by improving patient care pathways.

### Limitations

In this study only two traditional healers were interviewed, and all patients had advanced glaucoma whose views and experiences may differ from those with less advanced disease. All participants came from northeast Nigeria and the findings may not be applicable in other regions of the country where levels of education and socio-economic status are higher and where there are religious and cultural differences. Analysis was undertaken using translations of the transcripts and some nuances in meaning may have been lost, but the mother tongue of the first author is Hausa.

## Conclusions

Multiple interventions and approaches are required to reduce glaucoma blindness, the “silent thief of sight”, in Africa. Important elements include increasing awareness using locally derived terms so that patients present earlier; improving primary eye care for earlier detection and referral; improving the education of glaucoma patients about their disease and their role in controlling it; improving the quality, capabilities and efficiency of services and the attitudes of staff; ensuring the availability of affordable eye drops and teaching patients or their carers how to instil them, and evaluating interventions appropriate to Africa to improve adherence to topical medication, acceptance of surgery and long term follow up.

However, before demand for glaucoma services are generated in the community, interventions are required to improve the knowledge, acceptance and adherence to treatment amongst patients who present to eye departments in Africa with glaucoma. The findings from this study have been used to adapt a counselling technique known as motivational interviewing for glaucoma in the African context, which is being evaluated in a randomized controlled trial in ATBUTH [67].

## Ethics approval

Ethical approval was obtained from the Ethics Committees of the London School of Hygiene & Tropical Medicine and ATBUTH. After explaining the study they were given an information sheet or this was read out to them to obtain preliminary verbal consent. If they agreed to participate they were given information sheets to take home and a date to come back for the FGD. On the day of the discussion written consent was obtained by signature or thumbprint. Permission was explicitly obtained to record the discussions. The escort was also asked to give consent and allowed to participate. All participants were provided with refreshments and transport costs were reimbursed when needed.

## Availability of data

Transcripts from this study are available for sharing upon request. All identifying and confidential information about participants will however, be removed.

## Abbreviations

ATBUTH: Abubakar Tafawa Balewa University Teaching Hospital
BCPB: British Council for Prevention of Blindness
FGD: Focus group discussion
FM: Frequency Modulation
HIV/AIDS: Human Immune Deficiency Virus/Acquired Immune Deficiency
ICEH: International Centre for Eye Health
IDI: In-depth interview
USA: United States of America
